# Microstructured PVDF Film with Improved Performance as Flexible Infrared Sensor

**DOI:** 10.3390/s22072730

**Published:** 2022-04-02

**Authors:** Hongjian Guan, Weizhi Li, Ruilin Yang, Yuanjie Su, Hang Li

**Affiliations:** State Key Laboratory of Electronic Thin Films and Integrated Devices, University of Electronic Science and Technology of China, Chengdu 610054, China; 201822010103@std.uestc.edu.cn (H.G.); 202111050724@std.uestc.edu.cn (R.Y.); yjsu@uestc.edu.cn (Y.S.); 201922050129@std.uestc.edu.cn (H.L.)

**Keywords:** polyvinylidene fluoride, pyroelectric infrared detector, microstructure, flexible

## Abstract

Polyvinylidene fluoride (PVDF) is a very promising material for fabricating flexible infrared sensors due to its ferroelectricity as well as excellent flexibility and low fabrication cost. This work focuses on improving PVDF’s pyroelectric performance by creating microstructures in the film. Simulation results suggest that the pyroelectric response of PVDF film can be improved if micro groove, square-pit or sinusoidal patterns are created on the film surface, with the grooved film showing the best pyroelectric performance. Suggested by the simulation results, flexible PVDF samples with groove structure are prepared by casting the precursor solution on the mold with designed patterns. Measurement results demonstrate that the optimal microstructured PVDF film can improve its pyroelectric performance by as high as 146%, which is in good agreement with the simulations. This work provides an innovative way of achieving flexible infrared sensor devices with promoted performance based on pyroelectric polymers.

## 1. Introduction

**Figure 1:**
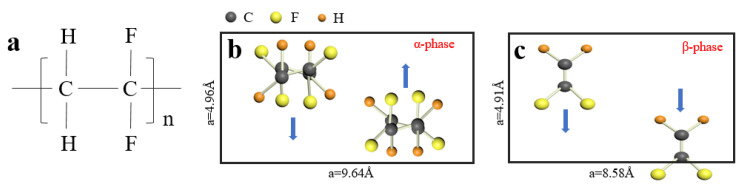
(**a**) Molecular structure of PVDF; (**b**) unit cell structure of non-ferroelectric α-phase PVDF; (**c**) unit cell structure of ferroelectric β-PVDF (the arrows indicate the dipole moments in the unit).

The pyroelectric sensor, the detection mechanism which is based on the pyroelectric effect of the sensitive material [1,2], is one of the most promising room temperature thermal sensors due to its potential of reaching the theoretical detection limit [3,4,5]. Because of its low price and stable technical performance, pyroelectric sensors are widely used in automatic control devices and are suitable for applications such as anti-theft alarms, automatic controls, etc. Pyroelectric sensitive materials can be divided into three categories: (a) ferroelectric single crystal, of which typical examples are lithium tantalite (LT) [6], triglyceride sulfate (TGS) [7], lithium niobate (LN) [8] and relaxor lead magnesium niobate lead titanate (PMNT) [9,10,11]; (b) ferroelectric ceramics, such as barium titanate (BT) [12,13], barium strontium titanate (BST) [14,15] and lead zirconate titanate (PZT) [16,17]; and (c) ferroelectric polymers, such as polyvinylidene fluoride (PVDF) [18,19,20] (Figure 1) and its copolymers [21,22,23]. Among these materials, ferroelectric single crystals and ceramics are widely used thanks to their large pyroelectric effect. However, the rigidness of ferroelectric crystals and ceramics makes them unable to be applied in wearable flexible electronics. In comparison, ferroelectric polymers especially PVDF-based polymers, are optimal candidates for wearable flexible electronics because of their excellent flexibility, low energy consumption, low-cost process [24,25,26,27,28,29] and even self-powering ability [30,31,32,33]. The biggest disadvantage of PVDF-based polymer, however, is its low pyroelectric coefficient and, consequently, low output signal, making it difficult to use them in practical applications like their inorganic counterparts.

To improve the pyroelectric performance of PVDF, tremendous work has focused on increasing its pyroelectric coefficient by incorporating a small quantity of ferroelectric inorganics such as BT [34], PZT [35,36,37], PT [38,39], calcium and lanthanum modified lead titanate (PCLT) [40], and even other active ingredients such as carbon nanotube (CNT) [41] and graphene [42]. This technique can improve the pyroelectric coefficient to some extent, but due to the large dielectric constant of the inorganic phase and the mismatch between the inorganic phase and PVDF matrix, the resulting composited film often has a large dielectric constant and loss, and therefore, the voltage and detectivity figures of merits (FOM) of the composites are usually even poorer than PVDF itself [43]. In addition, PVDF’s flexibility would deteriorate, and the film would become stiff and fragile because of the stiffness of the inorganic particles and defects caused by them.

In contrast to above-mentioned doping technique, some studies attempted to improve the ferroelectric performance of PVDF from other aspects. The preparation of PVDF nano- and micropillars by using anodic aluminum oxide membranes as templates are reviewed by Roman et al. [44], most studies surveyed in the review focus on the piezoelectric performance of the pillars, taking advantage of the 1D nanoconfinement to enhance the piezoelectric coefficient *d*_33_ of PVDF or P(VDF-TrFE) and, as a result, the energy harvesting performance of piezoelectric generators. Han et al. [45] adopted wet and dry etching technologies to fabricate microstructures directly on a thin β phase PVDF without losing its piezoelectricity. These microstructures are expected to form a soft, flexible and tactile interface with a sensation similar to human skin and cover robots’ entire body to ensure human safety when they unintentionally or deliberately come into contact or conflict with human beings. With the optimal etching condition, microstructured β PVDF film shows 50% of the original piezoelectricity. Zabek et al. [46] enhanced the infrared absorption of PVDF sensitive element by patterning the aluminum electrodes on PVDF film via a wet etching method, measured results demonstrated that the output signal was improved by about three-fold from the unpatterned counterpart.

In this work, pyroelectric PVDF’s performance is improved by patterning the film surface. A few different microstructures are designed with the influences of their geometric parameters on thermal performance of PVDF film being studied via finite element simulation. The results suggest that the film with optimized groove patterns has the best performance, the pyroelectric response of which is more than 2 times that of the non-structured PVDF film. According to the simulation results, PVDF samples with different groove structures are then fabricated by casting the PVDF solution on a metal plate with designed patterns. Measurement results demonstrate that the pyroelectric responses of the samples are in good agreement with the simulations. This work provides new ideas for the improvement of wearable sensor performance based on flexible pyroelectric film and shortens its distance toward practicality.

## 2. Finite Element Simulations and Discussion

**Figure 2:**
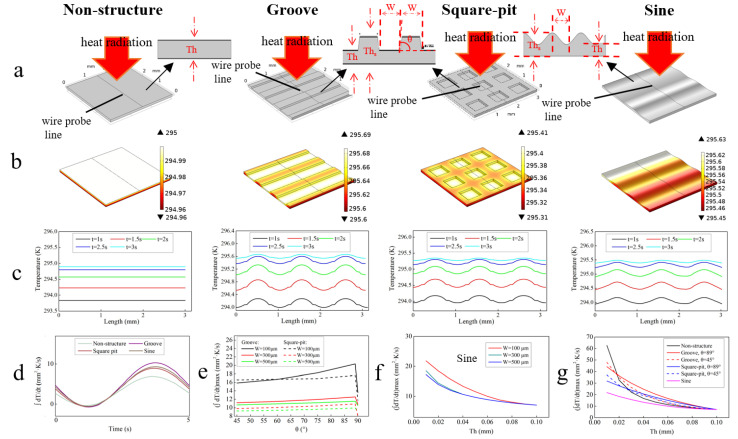
(**a**) Simulation models of non-structured, grooved, square-pitted and sinusoidal PVDF films. (**b**–**g**) Simulation results: (**b**) temperature distribution at *t* = 5 s for all models; (**c**) temperature distribution along wire probe lines at different times for all models; (**d**) ∫*dT*/*dt* over the film surface for all models; (**e**) influences of *θ* and *W* on (∫*dT*/*dt*)_max_ of the grooved and square-pitted films; (**f**) influences of *Th* and *W* on (∫*dT*/*dt*)_max_ of the sinusoidal films; (**g**) influences of *Th* on (∫*dT*/*dt*)_max_ of the structured and non-structured films.

As shown in Figure 2a, models of PVDF film with four different surface structures, i.e., flat (non-structured), grooved, square-pitted and sinusoidal surfaces are established. All films’ sizes are 3 mm × 3 mm. Both the thickness of the non-structured film (denoted as *Th* below) is 100 μm and the total thickness of three structured films (denoted as *Th_s_* below) is 100 μm. For the grooved (square-pitted) films, the bottom thickness of the grooves (pits) (which is also denoted as *Th* below in order to compare with the non-structured) is 50 μm. The angles between the sidewalls and the horizontal surface (denoted as *θ* below) are set at 45°. The width of the grooves (pits) and the distance between adjacent grooves (pits) (denoted as *W* below) are both 500 μm; for the sinusoidal model, half the sinusoid wavelength is also denoted as *W* and set at 500 μm. The relevant physical properties of PVDF are given in Table 1. The load condition is that alternating heat radiation is perpendicularly incident on the top surface, the radiation density of which is

(1) $$P=\left(100sin(0.4\pi t\right)+100){\;W/m}^{2},$$

where *t* is time. The bottom surface of the film is set to contact with dry air, of which the temperature is constant at 293.15 K. In addition, a wire probe is placed on the surface to obtain thermal information of points of interest at different times.

After simulation, the 3D diagrams of the surface temperature distribution of all models at different times are obtained, Figure 2b shows the results at the 5th second. It can be found that the maximum surface temperatures of the four films are 295 K, 295.69 K, 295.41 K and 295.63 K, respectively, which are, respectively, 1.75 K, 2.54 K, 2.26 K and 2.48 K higher than the initial temperature of 293.15 K.

Figure 2c gives surface temperature distribution along the wire probe lines at different times. It can be seen that for the non-structure film, surface temperature evenly distributes on the surface and temperature gradient only exists in the thickness direction. For the three structured films, in comparison, temperatures in the thin top surface areas are obviously higher than in the thick areas, whilst for the transitional areas between the thick and thin areas, i.e., the sidewalls, the temperatures are just between those of the bottom and top surfaces. These results simply originate from the fact that the area with smaller thickness has smaller thermal capacitance; thus, under the heat irradiation with the same intensity, the temperature rise in the thin areas will be bigger than in the thick ones. However, a higher temperature of the sensitive element does not necessarily indicate better pyroelectric performance, because according to the pyroelectric current expression

(2) $$i_{p}=pA_{s}\frac{dT}{dt},$$

(where *i_p_* is the pyroelectric current signal produced in the sensitive film, *p* the pyroelectric coefficient, *A_s_* the sensitive area and *T* the temperature of the element), it is the derivate of temperature with respect to time, i.e., *dT*/*dt*, that decides the pyroelectric signal of the sensitive element. Therefore, it is necessary to further investigate *dT*/*dt* on the films. However, unlike the non-structured film, the structured films have non-uniform surface temperature distribution, meaning *i_p_* is not uniform across the surface; therefore, *i_p_* of the total film can only be obtained by integrating current density *j_p_* across *A_s_*, i.e., Equation (2) should be substituted with

(3) $$i_{p}=\underset{A_{s}}{∯}j_{p}dxdy=p\underset{A_{s}}{∯}\frac{dT(x,y)}{dt}dxdy,$$

where *p* is assumed to be constant across the film. According to Equation (3), integral of *dT*/*dt* (denoted as ∫*dT*/*dt* below) over the film surface can be used to compare pyroelectric performances between different models, the results of which are given in Figure 2d. It shows that ∫*dT*/*dt* for all models are sinusoid-like functions of time, the maxima of which (denoted as (∫*dT*/*dt*)_max_ below) are 6.98, 10.63, 9.17 and 9.56 mm^2^·K/s, respectively, for the non-structure, sinusoidal, grooved and square-pitted films. These results indicate that microstructures in the PVDF film can improve its pyroelectric performance by 52.4%, 31.5% and 37.0%, respectively, for the three patterns.

Furthermore, the influences of *θ* and *W* on (∫*dT*/*dt*)_max_ of the grooved and square-pitted films, respectively, are studied, as shown in Figure 2e. It can be seen that (∫*dT*/*dt*)_max_ of both films slowly increase with *θ* as it changes from 45° to 89° and decreases with *W*. For example, when *W* = 100 μm, (∫*dT*/*dt*)_max_ of the grooved and the square-pitted films increases by 28% and 10%, respectively, as *θ* increases from 45° to 89°. As *θ* further increases from 89° to 90°, however, (∫*dT*/*dt*)_max_ of both structures sharply drop, but the dropping rate decreases as *W* increases. For instance, when *W* = 100 μm, (∫*dT*/*dt*)_max_ drops by about 33% for both structures, whereas as *W* increases to 500 μm, (∫*dT*/*dt*)_max_ only drops by about 10%. The influences of *Th* and *W* on (∫*dT*/*dt*)_max_ of the sinusoidal films are also studied as shown in Figure 2f. It shows that (∫*dT*/*dt*)_max_ decreases with both *Th* and *W*.

The influences of *Th* on (∫*dT*/*dt*)_max_ of all films (*W* and *Th*_s_ are set at 100 μm for all structured ones and *θ* is set at 45° or 89° for the grooved and square-pitted ones) are also investigated. As presented in Figure 2g, it can be found that large (∫*dT*/*dt*)_max_ can be achieved by reducing *Th*. However, clearly, when 20 μm < *Th* < 70 μm, (∫*dT*/*dt*)_max_ of the grooved and square-pitted PVDF is larger than that of the non-structured one (with the same *Th*) especially when *θ* is large. For example, when *Th* = 50 μm and *θ* = 89°, (∫*dT*/*dt*)_max_ of the grooved PVDF is 20.36 mm^2^·K/s, about 51% larger than that of the 50 μm-non-structured one (13.49 mm^2^·K/s), and roughly equal to that of the 30 μm-non-structured one (22.12 mm^2^·K/s). These results demonstrate that the grooved (or square-pitted) films can outperform the non-structured one even their total thickness of the former is larger than that of the latter. In comparison, (∫*dT*/*dt*)_max_ of the sinusoidal films is always smaller than that of the non-structured one at the same *Th*.

It needs to be pointed out that, although by further reducing the thickness of non-structured PVDF film the pyroelectric performance of the film can be improved [NASA], too small a thickness can easily lead to the electric breakdown of the film during the poling procedure, causing the poling to fail, hence PVDF film cannot be too thin. This might be why the thicknesses of commercial piezoelectric PVDF film are large enough. For example, PVDF film thicknesses from Measurement Specialties Inc. (now TE connectivity, the US), are around 30~50 μm or even larger [47]. While for traditional hard pyroelectric materials such as LT and PZT, too small a thickness will sharply increase technical difficulties and accordingly the cost; thus, commercial LT or PZT products are usually about 100μm thick. Therefore, creating microstructures in PVDF films to achieve better pyroelectric performance is of practical interest.

**Figure 3:**
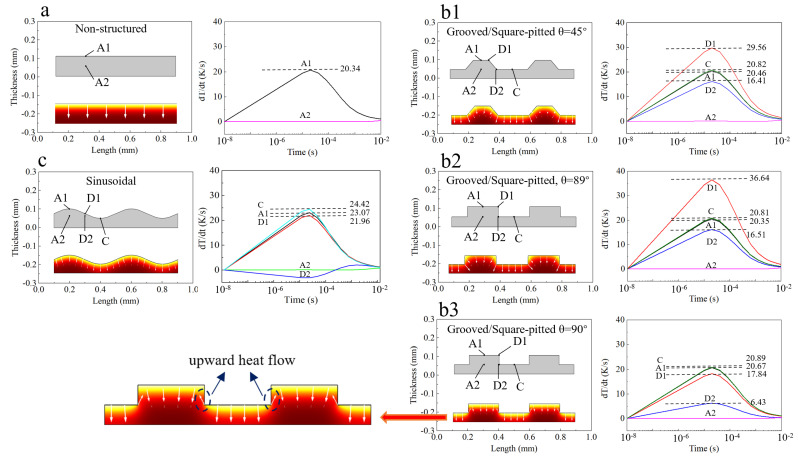
2D models, heat flow and dT/dt at points of interest for the study of underlying mechanism of improved performance of: (**a**) non. (**b1**–**b3**) Grooved. (**c**) Sinusoidal.

To further investigate the underlying mechanism behind the results in Figure 2, and for the sake of simplicity, five two-dimensional (2D) models as shown in Figure 3 are established to study the influence of different structures on the thermal properties of the films. Point probes are placed on a few points of interest in the films. The load condition is a constant heat flux of 100 W/m^2^ perpendicularly incident on the surface and time span of simulation is 1 × 10^−8^ s~1 × 10^−2^ s. It can be found that, for the unstructured film (Figure 3a), the heat flow only exists in the vertical direction and the largest *dT/dt* is 20.34 K/s which is located at point A1, and *dT/dt* at point A2 can be ignored. For the grooved (square-pitted) structures (Figure 3(b1–b3)), the largest *dT/dt*, appearing at D1, are 36.64 K/s when *θ* = 89° (Figure 3(b1)) and 29.56 K/s when *θ* = 45° (Figure 3(b2)), respectively, about 75% and 45% larger than that of the non-structured film. It can be also found that the heat flow not only exists in the top and bottom areas but in the sidewalls. In our opinion, it is these additional heat flow components that contribute to larger *dT*/*dt* in the two structures. Obviously, these heat flows are partially due to the partially upward sidewalls which are exposed to the incident heat irradiation; it is quite interesting that, although smaller *θ* can lead to more heat being received by the sidewall, *dT/dt* is still smaller than that when *θ* is larger, this result should be partially attributed to the decreased top horizontal surface area in the former. As *θ* increases to 90° (Figure 3(b3)), the largest *dT*/*dt* appears not at point D1 but point C, and is 20.81 K/s (the same as the maximum *dT*/*dt* at point C in Figure 3(b1,b2)). The maximum of *dT*/*dt* at D1 now is only 17.84 K/s, less than half that when *θ* = 89°. This sharp drop obviously is attributed to the now vertical sidewall which cannot receive any heat irradiation. An important consequence of this, as shown by the arrows in Figure 3(b3), is that there exist upward heat flow components at the sidewalls now, which will definitely compensate some heat loss at point D1, as a result, *dT*/*dt* at D1 drops a lot. For the sinusoidal structure (Figure 3c), on the other hand, since it has no steep sidewalls, the largest *dT/dt* is only 24.42 K/s and appears at point C.

According to these results, the groove structure (with optimized geometrics) shows the best performance and is relatively easy to build; it is then selected for real sample fabrication.

## 3. Fabrication and Test of Microstructured PVDF Films

The preparation process of the patterned PVDF film is shown in Figure 3a. A metal plate with 6 designed groove patterns is produced by a machining company; PVDF solution with mass fraction of 15% is prepared by dissolving PVDF powder (average molecular weight ≈ 534,000, Sigma Aldrich, St. Louis, MI, USA) in N-Methylpyrrolidone (NMP) solvent (99% purity, Kelong Chemicals, Chengdu, China). The solution is stirred at 70 °C for 2 h to achieve full dissolution and then placed in vacuum box for 1 h to remove bubbles. Then, the solution is slowly poured on the patterned metal plate, which was carefully cleaned before use, PVDF films are obtained by fully evaporating NMP solvent at 70 °C for 3 h, and then peeling them off the metal plate. Finally, in order to produce pyroelectricity in the samples, the samples, with electrodes prepared on both sides, are placed in a poling device (HYJH-8–10 KV, Huiyan New Material Technology Co., LTD, Xi’an, China) and poled in silicone oil at 90 °C with applied voltage of 900 V for 60 min, and then the samples are naturally cooled down with the poling voltage on until room temperature is reached.

**Figure 4:**
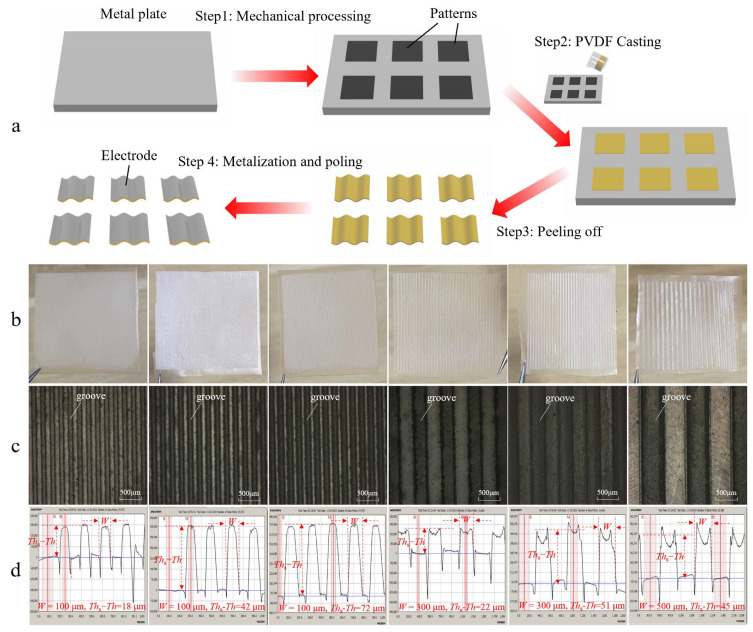
(**a**) Steps for microstructured PVDF films fabrication process; (**b**) photos of microstructured PVDF film samples (with the designed parameters given in the photos); (**c**) photos of morphologies of PVDF film samples; (**d**) measurement results of profiles of PVDF film samples (with the measured parameters given in the photos).

Photos of PVDF film samples and their morphologies are shown in Figure 4b,c, where grooves are clearly seen on the samples. The profiles of all samples are further measured with a surface profiler (XP-3, AmBioS Co. Ltd., Milpitas, CA, USA) as shown in Figure 4d. It demonstrates that samples with different groove geometrics are successfully prepared, but both the top and bottom surfaces are not smooth at all, which should be partially caused by the imperfect metal-plate patterns. Technique parameters of the casting process, such as solution concentration and evaporation temperature, may also play a role. These not-smooth-enough surfaces are likely to affect the effective electric field during thermal poling and, accordingly influence the pyroelectric performance of the samples. Table 2 summarizes the measured groove width (*W*) and depth (*Th*_s_−*Th*) of all structured samples.

**Figure 5:**
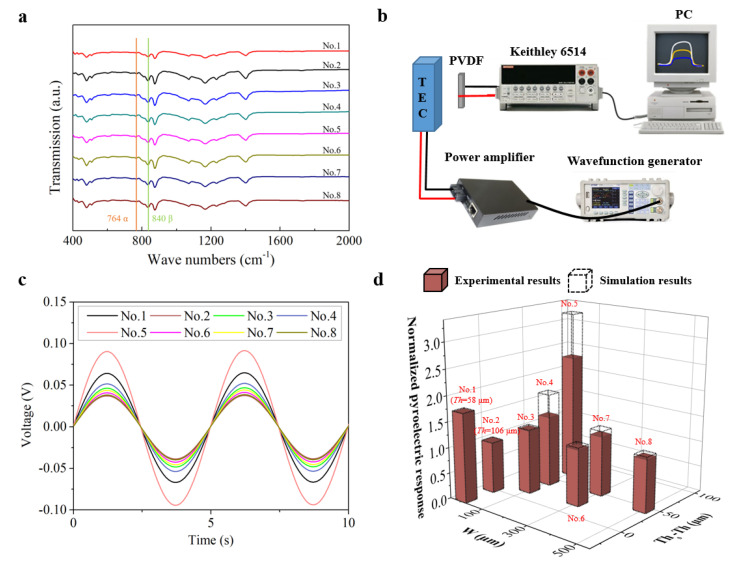
(**a**) FTIR spectra of PVDF samples. (**b**) Test platform for response performance of PVDF samples. (**c**) Real time response of PVDF samples. (**d**) Pyroelectric response results at different groove width (*W*) and depth (*Th_s_* − *Th*).

To further investigate whether molecular or phase change occurs in the samples after the groove patterns are introduced, Fourier transform infrared (FTIR) analysis are performed. As shown in Figure 5a, the typical α-phase characteristic peak at 764 cm^−1^ and β-phase characteristic peak at 840 cm^−1^ for each sample can be clearly distinguished. Compared with the unstructured films, the grooved samples do not introduce new characteristic peaks, indicating no molecular change occurred in the micro structured films. In addition, according to the two peak areas, β phase content percentage (*F*_β_) can be obtained [48] as given in the last line of Table 2. It can be found that eight samples have slightly different *F*_β_. In our opinion, these differences are most probably due to different poling effects during the thermal poling process of the samples. It is well known that PVDF films prepared by the normal technique, like the solution cast in this work, mainly comprise alpha phase (Figure 1b), which does not contribute to the ferroelectricity of PVDF film; therefore, thermal poling afterwards is very important because of its two effects: first is to convert alpha phase into beta phase (Figure 1b) and second is to align the randomly oriented dipoles in beta phase to the direction of poling electric field. Only after this poling process can PVDF become ferroelectric. However, because of the different surface roughness and morphology of the samples, effective poling electric field is a bit different among samples, therefore leading to a different poling effect and, accordingly, the slightly different *F*_β_ of the sample.

After above characterization, a platform to evaluate the samples’ pyroelectric performance is built as shown in Figure 5b. A thermo electric cooler (TEC) is used as the thermal source, the temperature of which is altered in a sinusoidal manner through a power-amplified sinusoidal signal via a wavefunction generator and power amplifier. The amplitude and period of TEC’s temperature are 5 °C and 5 s, respectively. The induced response of the sample is then collected by a Keithley 6514 electrometer and then sent to a PC. Figure 5c plots the actual response curves of the samples and Figure 5d compares the simulation and test responses. Table 2 summarizes all measured and simulated responses (the simulations are performed by using the measured *W*, *Th* and *Th*_s_, and = 60°).

It can be found that the response of the best grooved sample (No. 5) is more than two times that of the non-structured one which roughly has the same total thickness (No. 2), and also larger than the non-structured sample No. 1 which has a smaller thickness. It can be also found that there is a distinct difference (*R*_s_ − *R*_e_) between the measured and simulated responses, and the difference increases with the height of the groove (*Th*_s_ − *Th*). This is because, in the simulation, only *dT/dt* results can be obtained, but in reality, as described above, the poling process is vital to PVDF films’ pyroelectric response and the actual effect poling electric field in the sample is very complex due to the induced grooves rather than uniformly distributed as in the non-structured film. Intuitively, the electric field will become more complex as the height of the groove increases, which, in our opinion, will result in greater differences between the simulated response results and the experimental ones. As indicated in Table 2, sample No. 5 has the largest *Th*_s_ − *Th*, so does the discrepancy between the simulation and test results of this sample. Therefore, better methods such as micro electromechanical systems (MEMS) technology should be involved for sample fabrications, which are believed to achieve patterned samples with much better morphologies.

## 4. Conclusions

In summary, a novel method is presented to improve the performance of pyroelectric PVDF films by creating the microstructure on the surface of PVDF film. Simulation results indicate that by creating micro patterns such as grooves and pits on the surface of PVDF film, its pyroelectric performance can be greatly improved. The main contribution of the enhancement is believed to originate from the additional heat flow components appearing at the sidewalls of the patterns. Further simulation suggests that both the inclination angles of the sidewall and pattern width have an important impact on the film’s pyroelectric response. Based on the simulations, groove-structured PVDF samples are prepared by casting PVDF solution on a metal mold with different groove widths and depths. Test results show that the PVDF film’s pyroelectric performance is improved by 146% with optimized structure parameters, which is in agreement with the simulations. This work offers a cost-effective and reliable option for achieving a flexible PVDF film and infrared sensor device with a high performance.

## Figures and Tables

**Table 1:** Physical properties of PVDF.

Material	Density (kg/m^3^)	Thermal Conductivity (W/(m·K))	Specific Heat Capacity (J/(kg·K))
PVDF	1780	0.15	1314

**Table 2:** Measured parameters (the meaning of *W*, *Th* and *Th*_s_ can be referred to Figure 1, *R*_e_—normalized experimental response results, *F*_β_—β phase content) and normalized simulation response results (*R*_s_) of samples.

Parameters	Non-Structured Samples		Grooved Samples					
	No. 1	No. 2	No. 3	No. 4	No. 5	No. 6	No. 7	No. 8
*W* (μm)	-	-	100	100	100	300	300	500
*Th* (μm)	58	106	90	79	48	90	81	92
*Th*_s_ (μm)	-	-	108	121	120	112	132	127
*Th*_s_ − *Th* (μm)	-	-	18	42	72	22	51	45
*R*_s_	1.74	1	1.27	1.84	3.31	1.14	1.29	1.08
*R*_e_	1.70	1	1.28	1.43	2.46	1.14	1.17	1.07
*R*_s_ − *R*_e_	0.04	0	−0.01	0.41	0.85	0	0.12	0.01
*F*_β_	74.36%	73.31%	66.58%	68.90%	70.15%	74.43%	76.60%	80.24%
